# Nursing research on physical, relational and psychosocial care for older people in Germany: protocol for a mapping review guided by the Fundamentals of Care Framework

**DOI:** 10.1186/s13643-026-03191-0

**Published:** 2026-05-08

**Authors:** Ozlem Koseoglu Ornek, Franziska Jagoda, Claudia Dinand, Dominique Autschbach, Manuela Malek, Margareta Halek

**Affiliations:** School of Nursing Science, Witten/Herdecke University, Alfred-Herrhausen-Straße 50, Witten, 58455 Germany

**Keywords:** Fundamentals of care, Basic care, Nursing, Nursing care, Older people, Older adults, Aged, Mapping review, The Fundamentals of Care Framework

## Abstract

**Background:**

The number of older people in Germany has risen steadily in recent decades. One in four people is now aged 65 or over. As people age, their health problems tend to increase, as do their fundamental care needs. Nurses play a key role in addressing these needs through a holistic approach. To fulfil this responsibility effectively, it is necessary to examine existing nursing research on the fundamentals of care for older people and to identify gaps in the current evidence base. Therefore, we plan to conduct a mapping review with the aim of mapping the extent, range and nature of nursing research activities on the fundamentals of care, as defined in the physical, psychosocial and relational components of the Integration of Care dimension of the Fundamentals of Care Framework for older people in Germany.

**Methods:**

We will search the electronic databases PubMed/MEDLINE, CINAHL, CareLit and GeroLit, the catalogue of the German Federal Ministry of Research, Technology and Space and the German National Library for publications on nursing research based on the Integration of Care dimension of the Fundamentals of Care Framework among older people (≥ 65 years). There will be no time limit. We will include studies published in English and German. Initial screening of the first ten per cent of titles and abstracts and other stages will be carried out by two independent researchers. This process will be repeated until full agreement between the researchers. Any discrepancies will be resolved with consultation of a third reviewer. Results will be reported in a narrative synthesis and complemented by tabular and numerical presentations.

**Discussion:**

To the best of our knowledge, this mapping review will be the first to provide an overview of current nursing research on the fundamentals of care for older people in Germany. The inclusion of German-language texts and the absence of time limits in this review are intended to complement previous reviews. The planned mapping review will also identify the evidence gap in research in this area and contribute to the determination of future scientific research in Germany. Consequently, the findings of the mapping review could be of great interest to nurses and other health professionals for evidence-based practice, research and educational programmes. In addition, the data can be used to develop a programme for the provision of age-friendly and caring living conditions for older people in the future.

**Systematic review registration:**

The protocol was registered with Open Science Framework (osf.io/9e3uv).

**Supplementary Information:**

The online version contains supplementary material available at 10.1186/s13643-026-03191-0.

## Background

Globally, the number of older people has increased steadily over time for a number of reasons, including improvements in medical treatment and quality of life [1–3]. As projected by the World Health Organization, the global population of older individuals is anticipated to reach 2.1 billion by 2050, representing a twofold increase from the current figure. It is estimated that approximately three-fourths of these older populations reside in low-income countries [4]. However, the phenomenon of population ageing, defined as the gradual shift of a country’s population towards older ages [5], has already begun in high-income countries, including Germany [4, 6]. The latest data from the Federal Statistics Office (Statista) in Germany indicates that 22% (18.6 million) of the total population is aged 65 and over [7]. As evidenced by statistical data, the population of Germany is ageing, resulting in an increasing number of individuals requiring fundamental care [7]. In this context, fundamental care refers to the essential elements of nursing that support basic human needs. As people age, they often become less engaged in various aspects of life, including work, and may experience a range of health challenges, such as high blood pressure, chronic obstructive pulmonary disease, diabetes, dementia, cataracts, refractive errors, insomnia, loneliness, depression, hearing loss, and immobility [4, 8–12]. In addition, older people may be subjected to discrimination [13] and ageism [14], may be ignored, and may experience disrespectful behaviour, including negative attitudes and approaches that perceive them as an economic burden on social and health systems due to their age [15, 16]. Therefore, as people age, their frailty increases, and their need for comprehensive fundamentals of care services, encompassing physical, psychosocial, and relational aspects, becomes more pronounced [2, 4].

Fundamental care is a core component of nursing practice [17, 18], and occupies a pivotal role in the healthcare of older people [19, 20]. The concept of fundamental care was recognised many years ago by Florence Nightingale as comprising ‘essential elements in providing basic nursing care’ [21] and by the nurse theorist Henderson as ‘human needs’ [22]. Subsequently, a variety of other concepts were introduced into the nursing literature, including ‘personal care’, ‘self-care’, ‘activities of daily living care’, ‘essential care’, ‘basic nursing care’, and ‘basic care’ [23, 24]. However, these concepts were found to be inadequate for defining fundamentals of care due to their narrow focus on specific aspects of care [17, 18, 25, 26]. In addition, there appears to be a discrepancy between the perspectives of health staff, including nurses, and those of policymakers on the scope of fundamental care activities [17]. Furthermore, it is acknowledged that due to a shortage of staff, a lack of resources and an absence of effective organisational governance, nurses are compelled to prioritise other tasks over their primary responsibility of providing fundamentals of care [26–30]. For instance, in Germany, where economic pressures, rising care costs, and a shortage of nursing staff are increasingly prevalent, fundamental care for older people is largely delivered by family members, informal caregivers, and nursing assistants [31]. This indicates that policymakers and other key stakeholders may lack sufficient awareness of the importance, scope and impact of fundamentals of care. Such approaches and practices may lead to a decline in individuals’ quality of life, deterioration in mental well-being, and, in severe cases, increased mortality. In the long term, this may place an additional burden on the healthcare system [32, 33]. Therefore, the Fundamentals of Care (FOC) framework was developed, building upon previous theories and models of nursing care, to recognise the scope and importance of fundamentals of care, foster a common language among nurses, and elucidate the structure of comprehensive fundamentals of care to policy makers in a reasonable manner [18, 26].

**Fig. 1:**
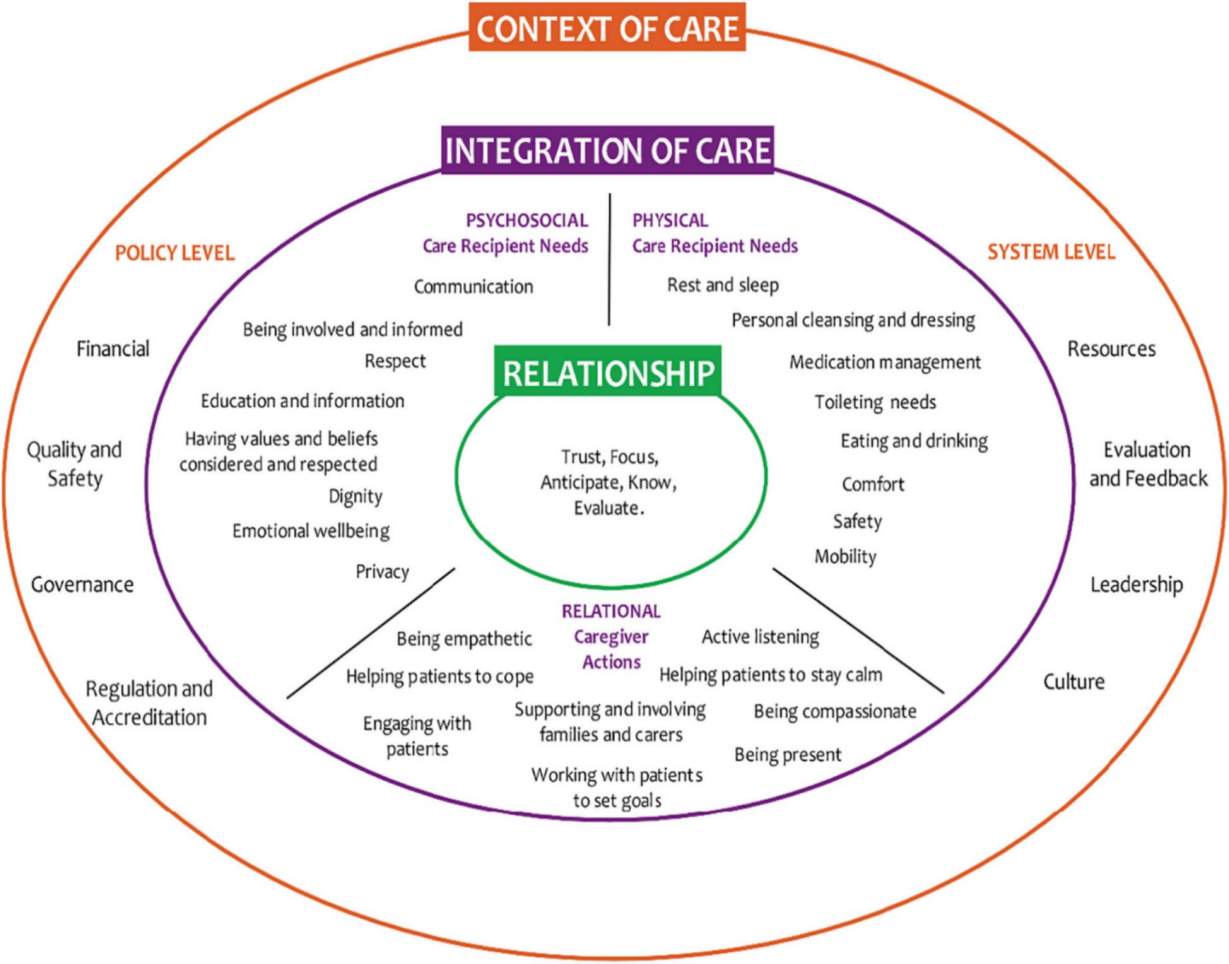
The Fundamentals of Care Framework. Image obtained from https://ilccare.org/the-fundamentals-of-care-framework/. Content within image derived from Feo, R., Conroy, T., Jangland. E., Muntlin Athlin, Å., Brovall, M., Parr, J., Blomberg, K., & Kitson, A. (2017). Towards a standardised definition for fundamental care: a modified Delphi study. *Journal of Clinical Nursing*, 27, 2285-2299. https://doi.org/10.1111/jocn.14247

The FOC framework was developed by Kitson et al. in 2010 [17]. It has been translated into many languages, including Norwegian, Swedish, French, Dutch and German [34, 35], and is widely used in nursing research [19, 36, 37], education [38, 39] and practice [24, 40]. It defines fundamental care as more comprehensive, broader, holistic and person-centred, and places the relationship at its centre compared to other nursing care theories and models [17, 18, 23, 25, 26, 41, 42]. It has three dimensions. The context of care dimension identifies external factors that influence the quality of care, including economics, regulations and accreditation, and leadership. The integration of care dimension describes the physical needs of people, such as mobility, personal hygiene, and dressing. It also describes the psychosocial needs of people, such as respect, emotional well-being, and relational fundamental care actions, such as being empathic and supportive. These approaches and care activities are effective in building a respectful, trusting, and positive relationship between the nurse and the patient and their family/relatives. The relationship dimension is therefore the core of this framework. It considers the patient and family/relatives as part of a whole [18, 26, 34] (see Fig. 1). Furthermore, the FOC is not just a framework for care; it also guarantees comprehensive and holistic care, illustrates the fundamentals of care activities and emphasises the crucial role of personal relationships as a prerequisite for optimal care for older people who are more likely to require multidimensional care [4, 20, 28, 43].

The need for fundamental care practices is increasing [4, 7]. Nurses, as key members of healthcare teams, must be adequately prepared through education and research to meet these needs [23, 38, 39, 42, 44]. To this end, it is essential to examine, compile, identify, catalogue and summarise the existing fundamental nursing research activities in a comprehensive and holistic manner with reference to the FOC framework. This will enable us to identify where research and implementation are required for optimal care of older people [45, 46]. A number of reviews have investigated nursing practices related to fundamentals of care for older people across various settings, including both clinical and home care contexts [19, 20]. For instance, Nordaunet et al. (2024) conducted a scoping review of studies written in English on fundamental nursing practices for older people in recent years [19]. Savoie et al. (2023) focused on all studies published in English and French in the first decade of the development of the FOC-framework to analyse the state of knowledge on fundamental care [27]. The systematic review by Richards et al. (2017) focused on some physical fundamentals of care needs [47]. These studies have made significant contributions to the nursing science literature on fundamentals of care [19, 20, 27, 47]. However, these reviews only include studies published in recent years. Of these, some have focused primarily on physical care practices [20, 47]. None have included research published in German, and none have been mapping reviews that examine the physical, psychosocial, and relational needs for older people in accordance with the FOC framework in the international field and in Germany. Therefore, we plan to conduct a mapping review with the clear objective of mapping the extent, range, and nature of nursing research on the fundamentals of care defined in the physical, psychosocial, and relational components of the Integration of Care dimension of the FOC framework for older people in Germany. This will be conducted without time restrictions and will contribute to addressing the existing knowledge gap. The findings will be used to inform the development of a national research programme aimed at improving the quality of life of care-dependent older people in the future.

The planned mapping review will specifically focus on answering the following scientific question: What is known about the extent, range, and nature of nursing research on Fundamentals of Care defined in the physical, relational and psychosocial components of the Integration of Care dimension of the FOC framework in Germany?

Sub-research questions are as follows:

- What evidence exists concerning the distribution of nursing research on the physical, relational, and psychosocial components of the Integration of Care dimension of FOC in Germany?
- What is the current state of knowledge regarding study designs and research methods used in nursing research on the Integration of Care dimension of FOC in Germany?
- What evidence exists concerning study settings and regional distribution (federal states in Germany) is reported in nursing research on the Integration of Care dimension of FOC in Germany?
- What is the distribution of funding sources for nursing research studies in Germany on the physical, relational, and psychosocial components of the Integration of Care dimension of the FOC framework, and which institutions provide this funding?
- What is the current state of knowledge about the evidence gap within nursing research on the physical, relational, and psychosocial components of the Integration of Care dimension of FOC?
- How has nursing research on the components of the Integration of Care dimension of the FoC framework changed over time in Germany?

## Method

We employed a mapping review design to address the research questions, systematically identify research gaps, and provide a comprehensive overview of the distribution of evidence within the field [45]. In the absence of a dedicated PRISMA extension for mapping reviews, PRISMA-ScR was selected as the most appropriate framework, in line with current methodological recommendations [46]. Therefore, this protocol is reported in accordance with the PRISMA-ScR (Preferred Reporting Items for Systematic Reviews and Meta-Analyses extension for Scoping Reviews) guideline [48] (see the PRISMA-ScR checklist in additional form 1).

Furthermore, the protocol is registered on the Open Science Framework (18 July 2024, osf.io/9e3uv). Any significant changes to the protocol will be reported in the mapping review publication. We used the RefHunter guide to plan, prepare and specify our research methodology [49].

### Eligibility criteria

The eligibility criteria of the included studies will be described based on the Population, Concept, and Context (PCC) framework to ensure a transparent and systematic selection process [50]. The concept of the PCC framework will be determined through the FOC-framework [18, 34] (see Fig. 1).

Population: People aged 65 years and over living in Germany will be included in the study irrespective of health status or diseases.

Concept: We will include empirical studies, reviews, study protocols, and grey literature, including conference outputs, theses and dissertations, project reports, and relevant books or book chapters, and descriptions of funded projects, provided that full-text information is accessible and that the source addresses nursing research on the physical and psychosocial care needs and relational care actions as defined within the Integration of Care dimension of the Fundamentals of Care Framework (see Fig. 1). Study protocols will be included to capture ongoing and emerging research and to provide a more comprehensive overview of the evidence landscape. Nursing research refers to studies conducted within the field of nursing science that focus on the physical, psychosocial, and relational aspects of care within the FOC framework. It also encompasses research undertaken by nursing scholars or by research teams with a formal affiliation to nursing.

Context: The review will include studies conducted in Germany on older people who were living in Germany at the time of data collection. Furthermore, studies included within the review will also be required to have been conducted in Germany.

Sources of evidence: We will include studies published in English and German conducted in Germany. No restrictions will be applied regarding study design, setting, or time frame. Studies published up to the end of July 2024, including e-publications ahead of print, will be included.

### Information sources

We will conduct a comprehensive search of the literature using the electronic databases PubMed/Medline, CINAHL, CareLit and GeroLit. We will also search the catalogue of the German Federal Ministry of Research, Technology and Space *(formerly the Federal Ministry of Education and Research)* of Germany for completed and ongoing projects. We will also search the catalogue of the German National Library (Katalog der Deutschen Nationalbibliothek) to ensure that we do not erroneously exclude research that was published as a thesis but not as an article, report or book. Furthermore, we will search by hand for potential studies in the journal ‘Pflege und Gesellschaft’, the journal of the German Society of Nursing Science, that have not been cited in the above-mentioned database.

If the full version of relevant publications is not available through these sources, we will contact the authors. Where the full text is not directly accessible via the authors, efforts will be made to obtain the documents through interlibrary loan services. If this is not possible, studies that remain inaccessible will be excluded from the review.

### Search strategies

We used a Peer Review of Electronic Search Strategy (PRESS) as a reference to create a reliable search strategy that identifies search errors and improves the selection of relevant search terms [51]. The three authors of this review (OKO, FJ, CD) searched relevant terms in the PubMed/Medline and CINAHL databases. Three independent researchers (OKO, FJ, CD) created a keyword pool by consensus. We then developed the search strategies for each electronic database using Medical Subject Headings (MeSH) and CINAHL Subject Headings, when applicable. We combined keywords and free-text search terms derived from the Integration of Care dimension of the FOC framework, including its components (physical, relational, and psychosocial needs) and their subcomponents [18, 34] and two additional search terms: nursing and older people (see additional form 2). Pilot searches were then conducted in PubMed/Medline and CINAHL database to test and refine the strategy.

### Study records

#### Data management

We will use EndNote X9 (Thomson Reuters), Rayyan [52], and MAXQDA 2024 (Clarivate Analytics, PA, USA) for data management.

#### Data collection and selection process

The above-mentioned databases will be searched using keywords in English and, where appropriate, in German. Subsequently, all publications in electronic databases will be transferred to the EndNote 21 programme (Clarivate Analytics), and any duplicate publications will be excluded. In the second stage, titles and abstracts of the identified publications will be screened using Rayyan, in accordance with the above-mentioned eligibility criteria [52]. A pilot phase will be conducted across all stages of mapping review in which the first ten percent of records will be independently assessed by two independent researchers. In cases of disagreement, an additional ten percent of records will be screened to further improve consensus between researchers. This process will be repeated until full agreement is achieved. Discrepancies will be resolved with consultation of a third researcher where necessary. Furthermore, the PRISMA flowchart presenting the screening process for the inclusion and exclusion of publications will be provided.

#### Data charting process

A structured and comprehensive data charting form has been developed to systematically extract relevant information from all included sources of evidence/information, based on the FOC framework’s predefined components and subcomponents [17, 25, 53]. Developed collaboratively by the review team, it specifies the variables to be extracted. The form will be piloted on a subset of included studies and iteratively refined to ensure clarity and consistency prior to full data charting (see Additional Form 3).

Charting variables will be aligned with the study aims and research questions. Calibration exercises among the review team will be conducted to ensure consistent interpretation and application of the charting criteria. The charting process will systematically map study outcomes and phenomena of interest to the Integration of Care dimension and its subcomponents, including physical care needs, psychosocial care needs, and relational care actions within the FOC framework.

The charting form will be iteratively refined throughout the process, with all modifications documented along with their rationale.

#### Data items

Data extraction will involve the systematic collection of predefined study characteristics from all included sources using a structured data charting form (see Additional Form 3). Extracted items will include study characteristics such as author(s), year of publication, study aim, research questions, study design, research methods, setting, geographical location, the language of publication, measurements, study population, sample characteristics, and type of publication (e.g. empirical study, review, protocol, or grey literature).

Additional items will capture the disciplinary and/or professional background of the authors, funding source (if reported). All data will be extracted in a standardised format to ensure consistency across studies.

### Risk of bias and quality assessment

A formal quality or risk of bias assessment will not be conducted in this mapping review. This decision aligns with the methodological aim of mapping reviews, which is to systematically identify, classify, and describe the distribution of evidence within a field rather than to evaluate the effectiveness or methodological quality of individual studies [45]. Therefore, there is no quality or risk of bias assessment of the included literature [48].

### Synthesis of results

Charted and extracted data will be synthesised using descriptive and narrative synthesis methods appropriate for a mapping review. Quantitative data will be summarised using frequencies and proportions to describe the distribution of included studies across the physical, psychosocial, and relational components of the Integration of Care dimension of the FOC framework. This will provide an overview of the characteristics of each included study as outlined above.

Studies will be coded and categorised using a structured coding framework developed a priori based on the FOC Integration of Care dimension, supported by MAXQDA 2024 (see Additional Form 4). Coding will be conducted in two stages based on the FOC framework’s predefined components/subcomponents [17, 25, 53]: initial classification based on study aims and research questions, followed by refinement based on reported outcomes and phenomena of interest to ensure alignment between conceptual intent and empirical findings. Studies may be mapped to multiple categories when addressing more than one care component or subcomponent.

The components and subcomponents of the Integration of Care dimension of FOC will serve as analytical anchors for component-level mapping and, where appropriate, for more granular categorisation (see Additional Form 4). Studies will be synthesised according to study characteristics and relevant variables described above to facilitate comparison across evidence types, with temporal trends analysed to identify changes in the evidence base over time.

Findings will be presented using narrative synthesis, summary tables, and visual mapping techniques (e.g. charts and diagrams) to illustrate the evidence landscape, identify clusters of research activity, and highlight gaps across domains of care.

## Discussion

This mapping review will provide a comprehensive description of the extent, range, and nature of nursing research based on the ‘integration of care’ dimension of the FOC framework for older people in Germany. It will also offer a detailed summary of nursing research conducted to date on the physical, psychosocial, and relational care needs of this population. It will also provide insight into the distribution of nursing research within this dimension and complement previous reviews by Savoie (2022) [27] and Nordaunet 2024 [19] by including German texts and not imposing time limitations.

We foresee a few strengths and limitations of our review. One of the key strengths of the planned mapping review is the use of the FOC framework [18, 34] as a definitive guide for the scope of Fundamentals of Care. The framework has been translated into different languages [34, 54] and is used in education research [38, 39, 55, 56], clinical research [36, 37, 40, 57, 58], and reviews [20, 27, 47, 59]. These studies prove that the framework is a reliable, systematic guide for creating a common language among nurses for delivering broad care needs of people, including older people, and in the examination of scientific gaps in the relevant topic [18, 19, 26, 60]. The framework-based mapping review will ensure that the authors’ perspectives and interpretations do not influence the data analysis. It will be conducted through a systematic and transparent approach [45]. Furthermore, the literature review revealed that all of the reviews on Fundamentals of Care examined studies published just after 2000 [19, 20, 27, 47]. However, it is evident that the background of physical care practices dates back to the 1860 s [21]. Therefore, we are confident that this mapping study will make a significant scientific contribution to the field by imposing no time restrictions. The inclusion of publications in both German and English also provides an advantage, as it may help reduce potential language bias, which can arise from the tendency for studies published in English to be more easily disseminated and indexed than those published in other languages [61]. A search of grey literature, including conference presentations and project abstracts from German electronic databases, will increase access to relevant research and help to mitigate the ‘file-drawer’ problem [62, 63]. Since grey literature is not typically peer-reviewed, there is a possibility that errors in the data may have been overlooked [64]. However, this is not expected to be a significant issue in the planned review given the methodological characteristics of a mapping review [45].

However, there are some potential limitations to this review. A variety of terms and approaches are used to define the fundamentals of care [17, 23, 25]. Therefore, to reduce the impact of this limitation, we conducted a detailed review of the relevant literature using keywords derived from the components of the FOC framework. This framework serves as an umbrella for fundamentals of care practices defined over time and provides a systematic structure for basic human care needs [23, 25, 26]. Another limitation is that, although we planned to use national and international databases that include relevant studies in the health field, not all research could be accessed, as some is published in book form. Therefore, we will address this limitation by manually searching the catalogue of the German Federal Ministry of Research, Technology and Space, where projects are widely published in Germany; the catalogue of the German National Library, which includes dissertations; and the journal *Pflege und Gesellschaft*, which publishes nursing research but is not indexed in the databases to be searched.

On the other hand, limiting the study to research conducted in Germany can be considered a limitation. However, Germany has substantial scientific expertise, and a large body of research is conducted in collaboration with international partners. In addition, Germany hosts many researchers from around the world at different stages of their academic careers. Therefore, we believe that conducting this review using international databases and English-language keywords will help mitigate the impact of this limitation [65].

Fundamentals of Care for older people have become an important public health issue in Germany [2, 4, 7]. It is imperative to protect the health of older people, meet their fundamental care needs, and ensure their quality of life. Therefore, a mapping review is planned to evaluate previous fundamental care research on the needs of older people in Germany and to set the stage for future studies by identifying gaps in the literature on fundamental care needs.

In addition, the findings may contribute to the development of programmes aimed at promoting age-friendly and care-supportive living conditions in the future. The results of this mapping review are therefore expected to be of particular relevance for nurses and other health professionals in evidence-based practice, research, and education. Finally, the findings will be disseminated through meetings, workshops, conferences, and publications.

## Supplementary Information

Additional file 1: PRISMA ScR Checklist. 

Additional file 2: Search Strings for the Databases. 

Additional file 3: Data charting form. 

Additional file 4: Coding Framework for Data Classification (FOC-Based).

## Data Availability

Not applicable.

## Abbreviations

WHO: World Health Organisation
FOC: The Fundamentals of Care Framework
PRISMA-ScR: The Preferred Reporting Items for Systematic Reviews and Meta-Analyses extension for Scoping Reviews
PCC: Population, Concept, Context
PRESS: Peer Review of Electronic Search Strategy
MeSH: Medical Subject Headings
