# Supplementary material for: Nursing research on physical, relational and psychosocial care for older people in Germany: protocol for a mapping review guided by the Fundamentals of Care Framework
Source: Syst Rev. 2026 May 8;15:160. doi: 10.1186/s13643-026-03191-0 (PMC13154650; doi:10.1186/s13643-026-03191-0)
Supplement: Supplementary file 2 — Additional file 2: Search Strings for the Databases. [file 13643_2026_3191_MOESM2_ESM.docx]

**Search Strings for the Databases**

1. **Pubmed/Medline**

Search ("nurs*"[Title/Abstract] OR "nursing care*"[Title/Abstract] OR "nurses"[MeSH Terms] OR "nursing"[MeSH Terms] OR "Nursing care"[MeSH Terms]) AND

("aged, 80 and over"[MeSH Terms] OR "aged"[MeSH Terms] OR "nursing homes"[MeSH Terms] OR "homes for the aged"[MeSH Terms] OR "geriatrics"[MeSH Terms] OR "elder*"[Title/Abstract] OR "aged"[Title/Abstract] OR "aged 80 and over"[Title/Abstract] OR "nursing home*"[Title/Abstract] OR "homes for the aged"[Title/Abstract] OR "geriatric*"[Title/Abstract] OR "elderly care*"[Title/Abstract] OR "elder care*"[Title/Abstract] OR "aged care*"[Title/Abstract] OR "old"[Title/Abstract] OR "seniors” [Title/Abstract] OR "senior” [Title/Abstract])

AND

("german*"[Title/Abstract] OR "germany"[MeSH Terms])

AND ("mobil*"[Title/Abstract] OR "range of motion, articular"[MeSH Terms] OR "exercis*"[Title/Abstract] OR "exercise"[MeSH Terms] OR "move*"[Title/Abstract] OR "movement"[MeSH Terms] OR "MOTION"[MeSH Terms] OR "Motion"[Title/Abstract] OR “Rest AND Sleep"[Title/Abstract] OR "Rest"[Title/Abstract] OR "Sleep"[Title/Abstract] OR "Rest"[MeSH Terms] OR "Sleep"[MeSH Terms] OR "relax*"[Title/Abstract] OR "restor*"[Title/Abstract] OR "Insomnia"[Title/Abstract] OR "NAP"[Title/Abstract] OR “Personal clean*”[Title/Abstract] OR “clean*”[Title/Abstract] OR “Dress*”[Title/Abstract] OR “Wash*”[Title/Abstract] OR “Bath*”[Title/Abstract] OR “Personal care” [Title/Abstract] OR “Body care”[Title/Abstract] OR “Basic care” [Title/Abstract] OR “Self care”[Title/Abstract] OR “Self care”[MeSH Terms] OR “Baths”[MeSH Terms] OR “Hygiene” [MeSH Terms] OR “Hygien*”[Title/Abstract] OR “Oral care” [Title/Abstract] OR “fundamental care*”[Title/Abstract] OR “Medica*”[Title/Abstract] OR “medical drug*”[Title/Abstract] OR “Medication manag*”[Title/Abstract] OR “medication administer*"[Title/Abstract] OR “pill” [Title/Abstract] OR “medication therapy manag*”[Title/Abstract] OR “medication therapy management”[MeSH Terms] OR "toilet*"[Title/Abstract] OR "eliminat*"[Title/Abstract] OR "excret*"[Title/Abstract] OR "voiding behav*"[Title/Abstract] OR "MICTURITION"[Title/Abstract] OR "urinat*"[Title/Abstract] OR "defecat*"[Title/Abstract] OR "stool*"[Title/Abstract] OR "defecation"[MeSH Terms] OR "urination"[MeSH Terms] OR "Eating and drinking"[Title/Abstract] OR "Eating"[Title/Abstract] OR "drink*"[Title/Abstract] OR "nutriti*"[Title/Abstract] OR "fluid*"[Title/Abstract] OR "oral intak*"[Title/Abstract] OR "feed*"[Title/Abstract] OR "oral hydrat*"[Title/Abstract] OR "Eating"[MeSH Terms] OR "drinking"[MeSH Terms] OR "alcohol drinking"[MeSH Terms] OR "nutritional stat*"[Title/Abstract] OR "Comfort"[Title/Abstract] OR "patient comfort*"[Title/Abstract] OR "patient satisf*"[Title/Abstract] OR "Patient satisfaction"[MeSH Terms] OR "Patient comfort"[MeSH Terms] OR “Safe*”[Title/Abstract] OR “Secur*”[Title/Abstract] OR “patient safe*”[Title/Abstract] OR “Patient safety” [MeSH Terms] OR “Safety” [MeSH Terms] OR "communicat*"[Title/Abstract] OR "communication"[MeSH Terms] OR "Health communication"[MeSH Terms] OR "health communicat*"[Title/Abstract] OR "conversation*"[Title/Abstract] OR "interact*"[Title/Abstract] OR "Exchange"[Title/Abstract] OR "involv*"[Title/Abstract] OR "inform*"[Title/Abstract] OR "participat*"[Title/Abstract] OR "patient participat*"[Title/Abstract] OR "patient participation"[MeSH Terms] OR "decision making, shared"[MeSH Terms] OR "decision making shared"[Title/Abstract] OR "social inclusi*"[Title/Abstract] OR "social inclusion"[MeSH Terms] OR "respect*"[Title/Abstract] OR "Esteem"[Title/Abstract] OR "cultur*"[Title/Abstract] OR "Stigma"[Title/Abstract] OR "Racism"[Title/Abstract] OR "discriminat*"[Title/Abstract] OR "Ageism"[Title/Abstract] OR "Social stigma"[Title/Abstract] OR "respect"[MeSH Terms] OR "Social stigma"[MeSH Terms] OR "Racism"[MeSH Terms] OR "Ageism"[MeSH Terms] OR "culture"[MeSH Terms] OR "educat*"[Title/Abstract] OR "inform*"[Title/Abstract] OR "health educat*"[Title/Abstract] OR "Health education"[MeSH Terms] OR "education"[MeSH Terms] OR "informed consent"[MeSH Terms] OR "informed consent*"[Title/Abstract] OR "Dignity"[Title/Abstract] OR "Honour"[Title/Abstract] OR "Honor"[Title/Abstract] OR "self respect*"[Title/Abstract] OR "respect self"[Title/Abstract] OR "self esteem*"[Title/Abstract] OR "self regard*"[Title/Abstract] OR "esteem self"[Title/Abstract] OR "concept self"[Title/Abstract] OR "Self Confidence"[Title/Abstract] OR "confidence self"[Title/Abstract] OR "Self important"[Title/Abstract] OR "Self concept"[Title/Abstract] OR "Self concept"[MeSH Terms] OR "Wellbeing"[Title/Abstract] OR "Well-Being"[Title/Abstract] OR "emotional wellbeing"[Title/Abstract] OR "emotion*"[Title/Abstract] OR "Quality of life"[Title/Abstract] OR "emotions"[MeSH Terms] OR "Quality of life"[MeSH Terms] OR "Psychological well-being"[MeSH Terms] OR " Psychological well-being "[Title/Abstract] OR "Personal satisfaction"[Title/Abstract] OR "Personal satisfaction"[MeSH Terms] OR "value*"[Title/Abstract] OR "belief*"[Title/Abstract] OR "religion*"[Title/Abstract] OR "spiritual*"[Title/Abstract] OR "cultur*"[Title/Abstract] OR "respect*"[Title/Abstract] OR "consider*"[Title/Abstract] OR "religion"[MeSH Terms] OR "culture"[MeSH Terms] OR "respect"[MeSH Terms] OR "Patient-Centered care"[MeSH Terms] OR "Patient-Centered care"[Title/Abstract] OR "Person-centered care"[Title/Abstract] OR "Privacy"[Title/Abstract] OR "privat*"[Title/Abstract] OR "Confidentiality"[Title/Abstract] OR "Personal space"[Title/Abstract] OR "Privacy"[MeSH Terms] OR "Confidentiality"[MeSH Terms] OR "Personal space"[MeSH Terms] OR "active listen*"[Title/Abstract] OR "Communication skill"[Title/Abstract] OR "listen*"[Title/Abstract] OR "attentive*"[Title/Abstract] OR "nurse patient relation*"[Title/Abstract] OR "Nurse-patient relations"[MeSH Terms] OR "empath*"[Title/Abstract] OR "sensitive*"[Title/Abstract] OR "compassion*"[Title/Abstract] OR "empathy"[MeSH Terms] OR "Attention"[MeSH Terms] OR "awareness"[MeSH Terms] OR "mindfulness"[MeSH Terms] OR "Nurse`s role"[MeSH Terms] OR "nurse s role*"[Title/Abstract] OR "Attention"[Title/Abstract] OR "aware*"[Title/Abstract] OR "mindful*"[Title/Abstract] OR "Being present"[Title/Abstract] OR "support*"[Title/Abstract] OR "involv*"[Title/Abstract] OR "famil*"[Title/Abstract] OR "friend*"[Title/Abstract] OR "Significant other"[Title/Abstract] OR "career*"[Title/Abstract] OR "Patient carer*"[Title/Abstract] OR "integrat*"[Title/Abstract] OR "caregiver*"[Title/Abstract] OR "caregivers"[MeSH Terms] OR "Social support"[MeSH Terms] OR "Social support"[Title/Abstract] OR "family involv*"[Title/Abstract] OR "patient participat*"[Title/Abstract] OR "patient engag*"[Title/Abstract] OR "Engagement patient"[Title/Abstract] OR "engag*"[Title/Abstract] OR "participat*"[Title/Abstract] OR "Patient participation"[MeSH Terms] "participat*"[Title/Abstract] OR "Patient participation"[MeSH Terms] OR "reassure*"[Title/Abstract] OR "support*"[Title/Abstract] OR "help*"[Title/Abstract] OR "enabl*"[Title/Abstract] OR "empower*"[Title/Abstract] OR "helping patient*"[Title/Abstract] OR "calm*"[Title/Abstract] OR "professional patient relation*"[Title/Abstract] OR "Professional-patient relations"[MeSH Terms] OR "support*"[Title/Abstract] OR "enabl*"[Title/Abstract] OR "empower*"[Title/Abstract] OR "coping behavior*"[Title/Abstract] OR "Cope"[Title/Abstract] OR "Coping"[Title/Abstract] OR "deal*"[Title/Abstract] OR "overcom*"[Title/Abstract] OR "coping skill*"[Title/Abstract] OR "Coping skills"[MeSH Terms] OR "compassion*"[Title/Abstract] OR "Self-compassion"[MeSH Terms] OR "sensitive*"[Title/Abstract] OR "self compassion*"[Title/Abstract] OR "goal*"[Title/Abstract] OR "Goals of care"[Title/Abstract] OR "patient care plan*"[Title/Abstract] OR "Nursing care plan"[Title/Abstract] OR "goals"[MeSH Terms] OR "Patient care planning"[MeSH Terms] OR "cooperation*"[Title/Abstract]) Filters: English, German, Medline, from 1000/1/1-2024/8/31

1. **CINAHL**

S1 AND S2 AND S3 AND S4

S1= MH Germany OR TI German* OR AB German*

S2= MH [Aged](javascript:XslPostBack('ctl00$ctl00$MainContentArea$MainContentArea$ctrlResults','meshDetail','index%7C5%24term%7CAged%24cmd%7CmeshDetail');) OR TI [Aged](javascript:XslPostBack('ctl00$ctl00$MainContentArea$MainContentArea$ctrlResults','meshDetail','index%7C5%24term%7CAged%24cmd%7CmeshDetail');) OR AB [Aged](javascript:XslPostBack('ctl00$ctl00$MainContentArea$MainContentArea$ctrlResults','meshDetail','index%7C5%24term%7CAged%24cmd%7CmeshDetail');) OR MH “[Hospitalization of Older Persons](javascript:XslPostBack('ctl00$ctl00$MainContentArea$MainContentArea$ctrlResults','meshDetail','index%7C2%24term%7CHospitalization%20of%20Older%20Persons%24cmd%7CmeshDetail');)“ OR TI “Hospitalization of Older Person*” OR AB “Hospitalization of Older Person*” OR MH “[Gerontologic Care](javascript:XslPostBack('ctl00$ctl00$MainContentArea$MainContentArea$ctrlResults','meshDetail','index%7C10%24term%7CGerontologic%20Care%24cmd%7CmeshDetail');)“ OR TI “[Gerontologic Care](javascript:XslPostBack('ctl00$ctl00$MainContentArea$MainContentArea$ctrlResults','meshDetail','index%7C10%24term%7CGerontologic%20Care%24cmd%7CmeshDetail');)*” OR AB “[Gerontologic Care](javascript:XslPostBack('ctl00$ctl00$MainContentArea$MainContentArea$ctrlResults','meshDetail','index%7C10%24term%7CGerontologic%20Care%24cmd%7CmeshDetail');)*” OR MH “[Aged, 80 and Over](javascript:XslPostBack('ctl00$ctl00$MainContentArea$MainContentArea$ctrlResults','meshDetail','index%7C1%24term%7CAged%2C%2080%20and%20Over%24cmd%7CmeshDetail');)” OR TI “[Aged, 80 and Over](javascript:XslPostBack('ctl00$ctl00$MainContentArea$MainContentArea$ctrlResults','meshDetail','index%7C1%24term%7CAged%2C%2080%20and%20Over%24cmd%7CmeshDetail');)“ OR AB “[Aged, 80 and Over](javascript:XslPostBack('ctl00$ctl00$MainContentArea$MainContentArea$ctrlResults','meshDetail','index%7C1%24term%7CAged%2C%2080%20and%20Over%24cmd%7CmeshDetail');)“ OR MH “[Nursing Homes](javascript:XslPostBack('ctl00$ctl00$MainContentArea$MainContentArea$ctrlResults','meshDetail','index%7C1%24term%7CNursing%20Homes%24cmd%7CmeshDetail');)“ OR TI “Nursing Homes*” OR AB “Nursing Home*” OR MH [Geriatrics](javascript:XslPostBack('ctl00$ctl00$MainContentArea$MainContentArea$ctrlResults','meshDetail','index%7C1%24term%7CGeriatrics%24cmd%7CmeshDetail');) OR TI Geriatric* OR AB Geriatric* OR TI elder* OR AB elder* OR TI “elderly care*” OR AB “elderly care*” OR TI “aged care*” OR AB “aged care*” OR TI old OR AB old OR TI senior* OR AB senior*

S3= TI nurs* OR AB nurs* OR MH nurse OR TI "nursing care*" OR AB "nursing care*" OR MH nurses

S4= MH "Physical Mobility" OR AB "Physical Mobilit*" OR TI "Physical Mobilit*" OR TI Mobilit* OR AB Mobilit* OR MH Mobility OR TI Movement* OR AB Movement* OR MH Movements OR MH Exercise OR TI Exercis* OR AB Exercis* OR MH Motion OR TI Motion* OR AB Motion* OR MH Physical OR TI Physical* OR AB Physical OR MH Performance OR TI Performanc* OR AB Performanc* OR MH Rest OR TI Rest* OR AB Rest* OR MH sleep OR TI Sleep* OR AB Sleep* OR MH “sleep and rest pattern*” OR AB Sleep and rest pattern*” OR TI Insomnia* OR AB Insomnia* OR TI Nap OR TI Naps AB Nap OR AB Naps OR MH [Dressing](javascript:XslPostBack('ctl00$ctl00$MainContentArea$MainContentArea$ctrlResults','meshDetail','index%7C1%24term%7CDressing%24cmd%7CmeshDetail');) OR TI Dress* OR AB Dress* OR MH Bathing OR TI Bath* OR AB Bath* OR MH “Self Care” OR TI “self care” OR “self care” OR MH “Self-care” OR TI “self-care*” OR “self-care*” OR MH hygiene OR TI Hygien* OR AB Hygien* OR MH “Personal care” OR TI “personal care*” OR AB “personal care*” OR MH Grooming OR TI Groom* OR AB groom* OR TI “personal clean* OR AB “personal clean* OR TI clean* OR AB clean* OR AB wasch* OR TI wasch OR TI Bath* OR AB Bath* OR TI “personal care*” OR AB “personal care*” OR TI “body care*” OR AB “body care*” OR TI “basic care*” OR AB “basic care*” OR TI “fundamental care*” OR “fundamental care*” OR MH “Medication management” OR TI “Medication manag*” OR AB “Medication manag*” OR MH “Medication treatment” OR TI “medication treatment*” OR AB “medication treatment*” OR MH “medication care” OR TI “medication care*” OR AB “medication care*” OR TI Medication* OR AB Medication* OR TI “Medicinal drug*” OR AB “medicinal drug*” OR TI pill* OR AB Pill* OR MH [Toileting](javascript:XslPostBack('ctl00$ctl00$MainContentArea$MainContentArea$ctrlResults','meshDetail','index%7C1%24term%7CToileting%24cmd%7CmeshDetail');) OR TI [Toileting](javascript:XslPostBack('ctl00$ctl00$MainContentArea$MainContentArea$ctrlResults','meshDetail','index%7C1%24term%7CToileting%24cmd%7CmeshDetail');)* AB [Toileting](javascript:XslPostBack('ctl00$ctl00$MainContentArea$MainContentArea$ctrlResults','meshDetail','index%7C1%24term%7CToileting%24cmd%7CmeshDetail');)* OR MH [Defecation](javascript:XslPostBack('ctl00$ctl00$MainContentArea$MainContentArea$ctrlResults','meshDetail','index%7C6%24term%7CDefecation%24cmd%7CmeshDetail');) OR TI [Defecation](javascript:XslPostBack('ctl00$ctl00$MainContentArea$MainContentArea$ctrlResults','meshDetail','index%7C6%24term%7CDefecation%24cmd%7CmeshDetail');)^*^ OR AB [Defecation](javascript:XslPostBack('ctl00$ctl00$MainContentArea$MainContentArea$ctrlResults','meshDetail','index%7C6%24term%7CDefecation%24cmd%7CmeshDetail');)* OR MH Urination OR TI Urination* OR AB Urination* OR TI Eliminat* OR AB Eliminat* OR TI Excret* OR AB Excret* OR TI “voiding behavior*” OR AB “voiding behavior*” OR TI mictur* OR AB mictur* OT TI defecat* OR AB defecat* OR TI stool* OR AB stool* OR MH [Eating](javascript:XslPostBack('ctl00$ctl00$MainContentArea$MainContentArea$ctrlResults','meshDetail','index%7C1%24term%7CEating%24cmd%7CmeshDetail');) OR TI Eating* OR AB Eating* OR MH Nutrition OR TI Nutrit* OR AB Nutrit* OR TI drink* OR AB drink* OR TI fluid* OR AB fluid* OR TI “oral intak*” OR AB “oral intak*” OR TI feed* OR AB feed* OR TI “oral hydrat*” OR AB “oral hydrat*” OR MH [Comfort](javascript:XslPostBack('ctl00$ctl00$MainContentArea$MainContentArea$ctrlResults','meshDetail','index%7C1%24term%7CComfort%24cmd%7CmeshDetail');) OR TI comfort* OR AB comfort* OR TI “patient comfort*” OR “ AB “patient comfort*” OR TI “patient satisf*” OR AB “patient satisf*” OR MH [Safety](javascript:XslPostBack('ctl00$ctl00$MainContentArea$MainContentArea$ctrlResults','meshDetail','index%7C1%24term%7CSafety%24cmd%7CmeshDetail');) OR TI Safe* OR AB safe* OR MH “patient safety” OR TI “patient safe*” OR AB “patient safe*” OR MH “Hand off” OR TI “hand off” OR AB “hand off” OR TI secur* OR AB secur* OR MH [Communication](javascript:XslPostBack('ctl00$ctl00$MainContentArea$MainContentArea$ctrlResults','meshDetail','index%7C1%24term%7CCommunication%24cmd%7CmeshDetail');) OR TI Communicat* OR AB Communicat* OR MH Conversation OR TI Conversat* OR AB Conversat* OR TI “Health Communicat*” OR AB “Health Communicat*” OR TI interact* OR AB interact* OR MH “[Decision Making, Shared](javascript:XslPostBack('ctl00$ctl00$MainContentArea$MainContentArea$ctrlResults','meshList','index%7C1%24term%7CDecision%20Making%2C%20Shared%24cmd%7CmeshList');)” OR TI “Decision Making, Shar*” OR AB “Decision Making, Shar*” OR MH “Social inclusion” OR TI “Social inclusi*” OR AB “Social inclusi*” OR MH “Patient participation” OR TI “Patient participat*” OR AB “Patient participat*” OR MH “Patient involvement” OR TI “Patient involve*” OR AB “Patient involve*” OR TI involv* OR AB involve* OR TI inform* OR AB inform* OR TI participat* OR AB participat* OR MH [Respect](javascript:XslPostBack('ctl00$ctl00$MainContentArea$MainContentArea$ctrlResults','meshDetail','index%7C1%24term%7CRespect%24cmd%7CmeshDetail');) OR TI [Respect](javascript:XslPostBack('ctl00$ctl00$MainContentArea$MainContentArea$ctrlResults','meshDetail','index%7C1%24term%7CRespect%24cmd%7CmeshDetail');)* OR AB [Respect](javascript:XslPostBack('ctl00$ctl00$MainContentArea$MainContentArea$ctrlResults','meshDetail','index%7C1%24term%7CRespect%24cmd%7CmeshDetail');)* OR MH [Culture](javascript:XslPostBack('ctl00$ctl00$MainContentArea$MainContentArea$ctrlResults','meshDetail','index%7C1%24term%7CCulture%24cmd%7CmeshDetail');) OR TI Cultur* OR AB Cultur* OR MH [Stigma](javascript:XslPostBack('ctl00$ctl00$MainContentArea$MainContentArea$ctrlResults','meshDetail','index%7C1%24term%7CStigma%24cmd%7CmeshDetail');) OR TI [Stigma](javascript:XslPostBack('ctl00$ctl00$MainContentArea$MainContentArea$ctrlResults','meshDetail','index%7C1%24term%7CStigma%24cmd%7CmeshDetail');)* OR AB [Stigma](javascript:XslPostBack('ctl00$ctl00$MainContentArea$MainContentArea$ctrlResults','meshDetail','index%7C1%24term%7CStigma%24cmd%7CmeshDetail');)* OR MH [Racism](javascript:XslPostBack('ctl00$ctl00$MainContentArea$MainContentArea$ctrlResults','meshDetail','index%7C1%24term%7CRacism%24cmd%7CmeshDetail');) OR TI [Racism](javascript:XslPostBack('ctl00$ctl00$MainContentArea$MainContentArea$ctrlResults','meshDetail','index%7C1%24term%7CRacism%24cmd%7CmeshDetail');) OR AB [Racism](javascript:XslPostBack('ctl00$ctl00$MainContentArea$MainContentArea$ctrlResults','meshDetail','index%7C1%24term%7CRacism%24cmd%7CmeshDetail');) OR MH Ageism OR TI Ageism OR AB Ageism OR MH [Discrimination](javascript:XslPostBack('ctl00$ctl00$MainContentArea$MainContentArea$ctrlResults','meshDetail','index%7C1%24term%7CDiscrimination%24cmd%7CmeshDetail');) OR TI Discriminat* OR AB Discriminat* OR TI Esteem* OR AB Esteem* OR MH “[Patient Education](javascript:XslPostBack('ctl00$ctl00$MainContentArea$MainContentArea$ctrlResults','meshDetail','index%7C12%24term%7CPatient%20Education%24cmd%7CmeshDetail');)” OR TI “patient education” OR AB “patient education” OR MH Education OR TI Educat* OR AB Educat* OR “[Health Education](javascript:XslPostBack('ctl00$ctl00$MainContentArea$MainContentArea$ctrlResults','meshDetail','index%7C1%24term%7CHealth%20Education%24cmd%7CmeshDetail');)” OR TI “[Health Education](javascript:XslPostBack('ctl00$ctl00$MainContentArea$MainContentArea$ctrlResults','meshDetail','index%7C1%24term%7CHealth%20Education%24cmd%7CmeshDetail');)” OR AB “[Health Education](javascript:XslPostBack('ctl00$ctl00$MainContentArea$MainContentArea$ctrlResults','meshDetail','index%7C1%24term%7CHealth%20Education%24cmd%7CmeshDetail');)” OR TI inform* OT AB inform* OR MH “[Self Concept](javascript:XslPostBack('ctl00$ctl00$MainContentArea$MainContentArea$ctrlResults','meshDetail','index%7C11%24term%7CSelf%20Concept%24cmd%7CmeshDetail');)” OR TI “[Self Concept](javascript:XslPostBack('ctl00$ctl00$MainContentArea$MainContentArea$ctrlResults','meshDetail','index%7C11%24term%7CSelf%20Concept%24cmd%7CmeshDetail');)*” AB “[Self Concept](javascript:XslPostBack('ctl00$ctl00$MainContentArea$MainContentArea$ctrlResults','meshDetail','index%7C11%24term%7CSelf%20Concept%24cmd%7CmeshDetail');)*” OR “[Human Dignity](javascript:XslPostBack('ctl00$ctl00$MainContentArea$MainContentArea$ctrlResults','meshDetail','index%7C3%24term%7CHuman%20Dignity%24cmd%7CmeshDetail');)” OR TI “Human Dignit*” OR AB “Human Dignit*” OR MH “Self-esteem” OR TI “Self-esteem*” OR AB “Self-esteem” OR TI “[Self-concept](javascript:XslPostBack('ctl00$ctl00$MainContentArea$MainContentArea$ctrlResults','meshDetail','index%7C11%24term%7CSelf%20Concept%24cmd%7CmeshDetail');)*” AB “[Self-concept](javascript:XslPostBack('ctl00$ctl00$MainContentArea$MainContentArea$ctrlResults','meshDetail','index%7C11%24term%7CSelf%20Concept%24cmd%7CmeshDetail');)*” OR TI Dignit* OR AB Dignit* OR TI Hono#r* AB Hono#r* OR TI “Self-regard*” OR AB “Self-regard*” OR TI “Self-confidence*” AB “Self-confidence*” OR TI “Self-important*” OR AB “Self-important*” OR MH “[Psychological Well-Being](javascript:XslPostBack('ctl00$ctl00$MainContentArea$MainContentArea$ctrlResults','meshDetail','index%7C2%24term%7CPsychological%20Well-Being%24cmd%7CmeshDetail');)” OR TI “[Psychological Well-Being](javascript:XslPostBack('ctl00$ctl00$MainContentArea$MainContentArea$ctrlResults','meshDetail','index%7C2%24term%7CPsychological%20Well-Being%24cmd%7CmeshDetail');)” AB “[Psychological Well-Being](javascript:XslPostBack('ctl00$ctl00$MainContentArea$MainContentArea$ctrlResults','meshDetail','index%7C2%24term%7CPsychological%20Well-Being%24cmd%7CmeshDetail');)” OR MH “Quality of life” OR TI “Quality of life*” OR AB “Quality of life*” OR MH “Well-being” OR TI “Well-being” OR AB ”Well-being” OR MH “Personal satisfaction” OR TI “Personal satisfact*” OR AB “Personal satisfact*” OR MH “[Emotions](javascript:XslPostBack('ctl00$ctl00$MainContentArea$MainContentArea$ctrlResults','meshDetail','index%7C5%24term%7CEmotions%24cmd%7CmeshDetail');)” OR TI Emotion* OR Emotion* OR MH “Religion and Religions” OR TI “[Religion and Religion*](javascript:XslPostBack('ctl00$ctl00$MainContentArea$MainContentArea$ctrlResults','meshDetail','index%7C2%24term%7CReligion%20and%20Religions%24cmd%7CmeshDetail');) OR AB “Religion and Religion*” OR MH Culture OR TI Culture OR AB Culture OR MH Respect OR TI Respect* OR AB Respect* OR MH [Spirituality](javascript:XslPostBack('ctl00$ctl00$MainContentArea$MainContentArea$ctrlResults','meshDetail','index%7C1%24term%7CSpirituality%24cmd%7CmeshDetail');) OR TI Spiritualit* AB Spiritualit* OR MH “person-centered care” OR TI person-centered care* OR AB “person-centered care* OR MH “[Privacy and Confidentiality](javascript:XslPostBack('ctl00$ctl00$MainContentArea$MainContentArea$ctrlResults','meshDetail','index%7C2%24term%7CPrivacy%20and%20Confidentiality%24cmd%7CmeshDetail');) TI “[Privacy and Confidential*”](javascript:XslPostBack('ctl00$ctl00$MainContentArea$MainContentArea$ctrlResults','meshDetail','index%7C2%24term%7CPrivacy%20and%20Confidentiality%24cmd%7CmeshDetail');) AB “[Privacy and Confidential*”](javascript:XslPostBack('ctl00$ctl00$MainContentArea$MainContentArea$ctrlResults','meshDetail','index%7C2%24term%7CPrivacy%20and%20Confidentiality%24cmd%7CmeshDetail');) OR MH “[Professional-Patient Relations](javascript:XslPostBack('ctl00$ctl00$MainContentArea$MainContentArea$ctrlResults','meshDetail','index%7C1%24term%7CProfessional-Patient%20Relations%24cmd%7CmeshDetail');)“ OR TI “[Professional-Patient Relations](javascript:XslPostBack('ctl00$ctl00$MainContentArea$MainContentArea$ctrlResults','meshDetail','index%7C1%24term%7CProfessional-Patient%20Relations%24cmd%7CmeshDetail');)*“ OR “AB “[Professional-Patient Relations](javascript:XslPostBack('ctl00$ctl00$MainContentArea$MainContentArea$ctrlResults','meshDetail','index%7C1%24term%7CProfessional-Patient%20Relations%24cmd%7CmeshDetail');)*“ OT TI Priva* OR TI Confidentiality OR AB Confidentiality OR TI “personal space OR AB “personal space OR MH “Active listening” OR TI “Active listening” OR AB “Active listening” OR MH “[Communication Skills](javascript:XslPostBack('ctl00$ctl00$MainContentArea$MainContentArea$ctrlResults','meshDetail','index%7C1%24term%7CCommunication%20Skills%24cmd%7CmeshDetail');)” OT “[Communication Skill*”](javascript:XslPostBack('ctl00$ctl00$MainContentArea$MainContentArea$ctrlResults','meshDetail','index%7C1%24term%7CCommunication%20Skills%24cmd%7CmeshDetail');) AB “[Communication Skill*”](javascript:XslPostBack('ctl00$ctl00$MainContentArea$MainContentArea$ctrlResults','meshDetail','index%7C1%24term%7CCommunication%20Skills%24cmd%7CmeshDetail');) OR MH “Nurse-Patient Relations” OR TI “Nurse-Patient Relation*”

AB “Nurse-Patient Relation*” OR MH “Listening” OR TI “Listening” OR AB “Listening” OR TI attentiveness OR AB attentive OR MH Empathy OR TI Empat* OR AB Empat* OR TI compassion* OR AB compassion* OR MH [Mindfulness](javascript:XslPostBack('ctl00$ctl00$MainContentArea$MainContentArea$ctrlResults','meshDetail','index%7C1%24term%7CMindfulness%24cmd%7CmeshDetail');) OR TI Mindful* OR AB Mindful* OR MH “[Nursing Role](javascript:XslPostBack('ctl00$ctl00$MainContentArea$MainContentArea$ctrlResults','meshDetail','index%7C1%24term%7CNursing%20Role%24cmd%7CmeshDetail');)“ OR TI “[Nursing Role](javascript:XslPostBack('ctl00$ctl00$MainContentArea$MainContentArea$ctrlResults','meshDetail','index%7C1%24term%7CNursing%20Role%24cmd%7CmeshDetail');)*” OR AB “[Nursing Role](javascript:XslPostBack('ctl00$ctl00$MainContentArea$MainContentArea$ctrlResults','meshDetail','index%7C1%24term%7CNursing%20Role%24cmd%7CmeshDetail');)*“ OR MH [Attention](javascript:XslPostBack('ctl00$ctl00$MainContentArea$MainContentArea$ctrlResults','meshDetail','index%7C1%24term%7CAttention%24cmd%7CmeshDetail');) OR TI Attenti* OR AB Attenti* OR MH [Family](https://www.ncbi.nlm.nih.gov/mesh/68005190) TI [Famil*](https://www.ncbi.nlm.nih.gov/mesh/68005190) OR AB [Famil*](https://www.ncbi.nlm.nih.gov/mesh/68005190) OR MH “Family involvement” OR TI “Family Involv*” AB “Family Involv*” OR MH “[Social Networks](javascript:XslPostBack('ctl00$ctl00$MainContentArea$MainContentArea$ctrlResults','meshDetail','index%7C6%24term%7CSocial%20Networks%24cmd%7CmeshDetail');)” OR TI “[Social Network*”](javascript:XslPostBack('ctl00$ctl00$MainContentArea$MainContentArea$ctrlResults','meshDetail','index%7C6%24term%7CSocial%20Networks%24cmd%7CmeshDetail');) AB “[Social Network*”](javascript:XslPostBack('ctl00$ctl00$MainContentArea$MainContentArea$ctrlResults','meshDetail','index%7C6%24term%7CSocial%20Networks%24cmd%7CmeshDetail');) OR MH [Caregivers](javascript:XslPostBack('ctl00$ctl00$MainContentArea$MainContentArea$ctrlResults','meshDetail','index%7C9%24term%7CCaregivers%24cmd%7CmeshDetail');) TI [Caregiver*](javascript:XslPostBack('ctl00$ctl00$MainContentArea$MainContentArea$ctrlResults','meshDetail','index%7C9%24term%7CCaregivers%24cmd%7CmeshDetail');) OR AB [Caregiver*](javascript:XslPostBack('ctl00$ctl00$MainContentArea$MainContentArea$ctrlResults','meshDetail','index%7C9%24term%7CCaregivers%24cmd%7CmeshDetail');) OR MH “Family Support” OR TI “Family Support*” OR AB “Family Support*” OR MH “Social support” OR TI “Social support*” AB “Social support*” OR TI Friend* OR AB Friend* OR TI Career* OR AB Career* OR TI “patient Care*” OR AB “patient Care*” OR MH “[Patient Participation](javascript:XslPostBack('ctl00$ctl00$MainContentArea$MainContentArea$ctrlResults','meshDetail','index%7C1%24term%7CPatient%20Participation%24cmd%7CmeshDetail');)“ OR TI “Patient Participa*” OR AB “Patient Participat*” OR TI Engag* OR AB Engag* OR TI “Engaging with patient*” OR AB “Engaging with patient*” OR MH “[Emotional Support](javascript:XslPostBack('ctl00$ctl00$MainContentArea$MainContentArea$ctrlResults','meshDetail','index%7C1%24term%7CEmotional%20Support%20%28Iowa%20NIC%29%24cmd%7CmeshDetail');)“ OR TI “[Emotional Support](javascript:XslPostBack('ctl00$ctl00$MainContentArea$MainContentArea$ctrlResults','meshDetail','index%7C1%24term%7CEmotional%20Support%20%28Iowa%20NIC%29%24cmd%7CmeshDetail');)*” OR Ab “[Emotional Support](javascript:XslPostBack('ctl00$ctl00$MainContentArea$MainContentArea$ctrlResults','meshDetail','index%7C1%24term%7CEmotional%20Support%20%28Iowa%20NIC%29%24cmd%7CmeshDetail');)*” OR MH “[Professional-Patient Relations](javascript:XslPostBack('ctl00$ctl00$MainContentArea$MainContentArea$ctrlResults','meshDetail','index%7C1%24term%7CProfessional-Patient%20Relations%24cmd%7CmeshDetail');)” OR TI “Professional-Patient Relation*” OR AB “Professional-Patient Relation*” OR MH “Patient rehabilitation” OR TI “Patient rehabilitant*” OR AB “Patient rehabilitant*” OR TI reassure* OR AB reassure* OR TI support* OR AB support* OR TI help* OR AB help* OR TI enabl* OR AB enabl* OR TI empower* OR AB empower* OR TI Calm* OR AB Calm* OR MH [Coping](javascript:XslPostBack('ctl00$ctl00$MainContentArea$MainContentArea$ctrlResults','meshDetail','index%7C12%24term%7CCoping%24cmd%7CmeshDetail');) OR TI [Coping](javascript:XslPostBack('ctl00$ctl00$MainContentArea$MainContentArea$ctrlResults','meshDetail','index%7C12%24term%7CCoping%24cmd%7CmeshDetail');) AB [Coping](javascript:XslPostBack('ctl00$ctl00$MainContentArea$MainContentArea$ctrlResults','meshDetail','index%7C12%24term%7CCoping%24cmd%7CmeshDetail');) OR MH “Coping Support” OR TI “Coping Support*” OR AB “Coping Support*” OR MH “Coping Component” OR TI “Coping Component*” OR AB “Coping Component*” OR TI support* OR AB support* OR TI enable* OR AB enable* OR TI Empower* OR AB empower* OR TI “coping behavio#r* OR AB “coping behavio#r* OR TI Cope* OR AB Cope* OR TI deal* OR AB deal* OR TI overcom* OR AB overcome* OR TI “coping skill*” OR AB “coping skill*” OR MH [Compassion](javascript:XslPostBack('ctl00$ctl00$MainContentArea$MainContentArea$ctrlResults','meshDetail','index%7C1%24term%7CCompassion%24cmd%7CmeshDetail');) OR TI Compass* OR AB Compass* OR MH “[Self-Compassion](https://www.ncbi.nlm.nih.gov/mesh/2101031)“ OR TI “Self-Compass*” OR AB “Self-Compass*” OR TI “sensitive* OR AB sensitive* OR MH “[Goal-Setting](javascript:XslPostBack('ctl00$ctl00$MainContentArea$MainContentArea$ctrlResults','meshDetail','index%7C6%24term%7CGoal-Setting%24cmd%7CmeshDetail');)“ OT TI “[Goal-Setting](javascript:XslPostBack('ctl00$ctl00$MainContentArea$MainContentArea$ctrlResults','meshDetail','index%7C6%24term%7CGoal-Setting%24cmd%7CmeshDetail');)*” OR AB “[Goal-Setting](javascript:XslPostBack('ctl00$ctl00$MainContentArea$MainContentArea$ctrlResults','meshDetail','index%7C6%24term%7CGoal-Setting%24cmd%7CmeshDetail');)*” OR MH “Mutual Goal Setting” OR TI “Mutual Goal Setting*” OR AB “Mutual Goal Setting*” OR TI Goal* OR AB Goal* OR TI Cooperat* OR AB Cooperat*

Filters applied: **Limiters** - Publication Date: 19000101-20241231 **Expanders** - Apply equivalent subjects **Narrow by Language:**- german, english **Search modes** - Boolean/Phrase

1. **The Federal Ministry of Education and Research (FöKAT)**

%Pflege%, no filters applied.

1. GeroLit:  Alle Wörter= [ALL]

[[ALL]**Pflege**](https://vzlbs2.gbv.de/DB=41/SET=4/TTL=3/NXT?SET=4) und [ALL] Deutschland und [ALL] Mobilität

[[ALL]**Pflege**](https://vzlbs2.gbv.de/DB=41/SET=4/TTL=3/NXT?SET=4) und [ALL] Deutschland und [ALL] Bewegung

[[ALL]**Pflege**](https://vzlbs2.gbv.de/DB=41/SET=4/TTL=3/NXT?SET=4) und [ALL] Deutschland und [ALL] Ruhe

[[ALL]**Pflege**](https://vzlbs2.gbv.de/DB=41/SET=4/TTL=3/NXT?SET=4) und [ALL] Deutschland und [ALL] Schlaf

[[ALL]**Pflege**](https://vzlbs2.gbv.de/DB=41/SET=4/TTL=3/NXT?SET=4) und [ALL] Deutschland und [ALL] Schlaflosigkeit

[[ALL]**Pflege**](https://vzlbs2.gbv.de/DB=41/SET=4/TTL=3/NXT?SET=4) und [ALL] Deutschland und [ALL] Körperpflege

[[ALL]**Pflege**](https://vzlbs2.gbv.de/DB=41/SET=4/TTL=3/NXT?SET=4) und [ALL] Deutschland und [ALL] kleiden

[[ALL]**Pflege**](https://vzlbs2.gbv.de/DB=41/SET=4/TTL=3/NXT?SET=4) und [ALL] Deutschland und [ALL] Körperhygiene

[[ALL]**Pflege**](https://vzlbs2.gbv.de/DB=41/SET=4/TTL=3/NXT?SET=4) und [ALL] Deutschland und [ALL] Medikation,

[[ALL]**Pflege**](https://vzlbs2.gbv.de/DB=41/SET=4/TTL=3/NXT?SET=4) und [ALL] Deutschland und [ALL]  „Medikamentöse Behandlung“ ,

[[ALL]**Pflege**](https://vzlbs2.gbv.de/DB=41/SET=4/TTL=3/NXT?SET=4) und [ALL] Deutschland und [ALL] Medikamentenmanagement

[[ALL]**Pflege**](https://vzlbs2.gbv.de/DB=41/SET=4/TTL=3/NXT?SET=4) und [ALL] Deutschland und [ALL] Ausscheiden

[[ALL]**Pflege**](https://vzlbs2.gbv.de/DB=41/SET=4/TTL=3/NXT?SET=4) und [ALL] Deutschland und [ALL] „Essen und Trinken“

[[ALL]**Pflege**](https://vzlbs2.gbv.de/DB=41/SET=4/TTL=3/NXT?SET=4) und [ALL] Deutschland und [ALL] Ernährung

[[ALL]**Pflege**](https://vzlbs2.gbv.de/DB=41/SET=4/TTL=3/NXT?SET=4) und [ALL] Deutschland und [ALL] Flüssigkeit

[[ALL]**Pflege**](https://vzlbs2.gbv.de/DB=41/SET=4/TTL=3/NXT?SET=4) und [ALL] Deutschland und [ALL] Komfort

[[[ALL]**Pflege**](https://vzlbs2.gbv.de/DB=41/SET=4/TTL=3/NXT?SET=4) und [ALL] Deutschland und ALL] Zufriedenheit

[[ALL]**Pflege**](https://vzlbs2.gbv.de/DB=41/SET=4/TTL=3/NXT?SET=4) und [ALL] Deutschland und [ALL] Sicher

[[ALL]**Pflege**](https://vzlbs2.gbv.de/DB=41/SET=4/TTL=3/NXT?SET=4) und [ALL] Deutschland und [ALL] Kommunikation

[[ALL]**Pflege**](https://vzlbs2.gbv.de/DB=41/SET=4/TTL=3/NXT?SET=4) und [ALL] Deutschland und [ALL] Interaktion

[[ALL]**Pflege**](https://vzlbs2.gbv.de/DB=41/SET=4/TTL=3/NXT?SET=4) und [ALL] Deutschland und [ALL] Beteiligen

[[ALL]**Pflege**](https://vzlbs2.gbv.de/DB=41/SET=4/TTL=3/NXT?SET=4) und [ALL] Deutschland und [ALL] Respekt

[[ALL]**Pflege**](https://vzlbs2.gbv.de/DB=41/SET=4/TTL=3/NXT?SET=4) und [ALL] Deutschland und [ALL] Rassismus

[[ALL]**Pflege**](https://vzlbs2.gbv.de/DB=41/SET=4/TTL=3/NXT?SET=4) und [ALL] Deutschland und [ALL] Altersdiskriminierung

[[ALL]**Pflege**](https://vzlbs2.gbv.de/DB=41/SET=4/TTL=3/NXT?SET=4) und [ALL] Deutschland und [ALL] Edukation

[[ALL]**Pflege**](https://vzlbs2.gbv.de/DB=41/SET=4/TTL=3/NXT?SET=4) und [ALL] Deutschland und [ALL] Information

[[ALL]**Pflege**](https://vzlbs2.gbv.de/DB=41/SET=4/TTL=3/NXT?SET=4) und [ALL] Deutschland und [ALL] Würde

[[ALL]**Pflege**](https://vzlbs2.gbv.de/DB=41/SET=4/TTL=3/NXT?SET=4) und [ALL] Deutschland und [ALL] Wohlbefinden

[[ALL]**Pflege**](https://vzlbs2.gbv.de/DB=41/SET=4/TTL=3/NXT?SET=4) und [ALL] Deutschland und [ALL] Emotionen

[[ALL]**Pflege**](https://vzlbs2.gbv.de/DB=41/SET=4/TTL=3/NXT?SET=4) und [ALL] Deutschland und [ALL] Lebensqualität

[[ALL]**Pflege**](https://vzlbs2.gbv.de/DB=41/SET=4/TTL=3/NXT?SET=4) und [ALL] Deutschland und [ALL] Wert

[[ALL]**Pflege**](https://vzlbs2.gbv.de/DB=41/SET=4/TTL=3/NXT?SET=4) und [ALL] Deutschland und [ALL] Überzeugung

[[ALL]**Pflege**](https://vzlbs2.gbv.de/DB=41/SET=4/TTL=3/NXT?SET=4) und [ALL] Deutschland und [ALL] Privat

[[ALL]**Pflege**](https://vzlbs2.gbv.de/DB=41/SET=4/TTL=3/NXT?SET=4) und [ALL] Deutschland und [ALL] Zuhören

[[ALL]**Pflege**](https://vzlbs2.gbv.de/DB=41/SET=4/TTL=3/NXT?SET=4) und [ALL] Deutschland und [ALL] Kommunikationsfähigkeit

[[ALL]**Pflege**](https://vzlbs2.gbv.de/DB=41/SET=4/TTL=3/NXT?SET=4) und [ALL] Deutschland und [ALL] empathisch

[[ALL]**Pflege**](https://vzlbs2.gbv.de/DB=41/SET=4/TTL=3/NXT?SET=4) und [ALL] Deutschland und [ALL] Einfühlungsvermögen

[[ALL]**Pflege**](https://vzlbs2.gbv.de/DB=41/SET=4/TTL=3/NXT?SET=4) und [ALL] Deutschland und [ALL] Einfühlsam

[[ALL]**Pflege**](https://vzlbs2.gbv.de/DB=41/SET=4/TTL=3/NXT?SET=4) und [ALL] Deutschland und [ALL] Achtsamkeit

[[ALL]**Pflege**](https://vzlbs2.gbv.de/DB=41/SET=4/TTL=3/NXT?SET=4) und [ALL] Deutschland und [ALL] Unterstützen

[[ALL]**Pflege**](https://vzlbs2.gbv.de/DB=41/SET=4/TTL=3/NXT?SET=4) und [ALL] Deutschland und [ALL] "sich einlassen"

[[ALL]**Pflege**](https://vzlbs2.gbv.de/DB=41/SET=4/TTL=3/NXT?SET=4) und [ALL] Deutschland und [ALL] "Zusammenarbeit mit Patienten"

[[ALL]**Pflege**](https://vzlbs2.gbv.de/DB=41/SET=4/TTL=3/NXT?SET=4) und [ALL] Deutschland und [ALL] partnerschaft

[[ALL]**Pflege**](https://vzlbs2.gbv.de/DB=41/SET=4/TTL=3/NXT?SET=4) und [ALL] Deutschland und [ALL] beruhigen

[[ALL]**Pflege**](https://vzlbs2.gbv.de/DB=41/SET=4/TTL=3/NXT?SET=4) und [ALL] Deutschland und [ALL] hilfen

[[ALL]**Pflege**](https://vzlbs2.gbv.de/DB=41/SET=4/TTL=3/NXT?SET=4) und [ALL] Deutschland und [ALL] Bewältigung

[[ALL]**Pflege**](https://vzlbs2.gbv.de/DB=41/SET=4/TTL=3/NXT?SET=4) und [ALL] Deutschland und [ALL] Mitgefühl

[[ALL]**Pflege**](https://vzlbs2.gbv.de/DB=41/SET=4/TTL=3/NXT?SET=4) und [ALL] Deutschland und [ALL] Mitgefühlen

[[ALL]**Pflege**](https://vzlbs2.gbv.de/DB=41/SET=4/TTL=3/NXT?SET=4) und [ALL] Deutschland und [ALL] Ziel

[[ALL]**Pflege**](https://vzlbs2.gbv.de/DB=41/SET=4/TTL=3/NXT?SET=4) und [ALL] Deutschland und [ALL] Pflegeplan

Filters applied: **Limiters**  unscharfe Suche

1. CareLit

(TITEL=Pflege ODER TITEL=Krankenschwester) UND (TITEL=Deutschland) UND (TITEL=alt ODER TITEL=älter ODER TITEL=Geriatrie ODER TITEL=Senioren ODER TITEL=Altenheim)

(ABSTRACT=Pflege ODER ABSTRACT=Krankenschwester) UND (ABSTRACT=Deutschland) UND (ABSTRACT=alt ODER ABSTRACT=älter ODER ABSTRACT=Geriatrie ODER ABSTRACT=Senioren ODER ABSTRACT=Altenheim)

no filters applied.

1. **The catalogue of the German National Library**

Alle begriefe (woe all )=all letters

woe all "pflege*" and woe all "Mobilität*" and woe all "alt*"

woe all "pflege*" and woe all "Mobilität*" and woe all "älter*"

woe all "pflege*" and woe all "Mobilität*" and woe all "Geriatri*“

woe all "pflege*" and woe all "Mobilität*" and woe all "Senior*"

woe all "pflege*" and woe all "Beweg*" and woe all "alt*"

woe all "pflege*" and woe all "Beweg*" and woe all "älter*"

woe all "pflege*" and woe all "Beweg*" and woe all "Geriatri*"

woe all "pflege*" and woe all "Beweg*" and woe all "Senior*"

woe all "pflege*" and woe all " Körperpflege*" and woe all "alt*"

woe all "pflege*" and woe all "Körperpflege*" and woe all "älter*"

woe all "pflege*" and woe all " Körperpflege*" and woe all "Geriatri*"

woe all "pflege*" and woe all " Körperpflege*" and woe all "Senior*"

woe all "pflege*" and woe all "kleid*" and woe all "alt*"

woe all "pflege*" and woe all "kleid*" and woe all "Senior*"

woe all "pflege*" and woe all "körperhygien*" and woe all "alt*"

woe all "pflege*" and woe all "körperhygien*" and woe all "alter*"

woe all "pflege*" and woe all " körperhygien *" and woe all "Geriatri*"

woe all "pflege*" and woe all " körperhygien *" and woe all "Senior*"

woe all "pflege*" and woe all "medika*" and woe all "alt*"

woe all "pflege*" and woe all "medika*" and woe all "älter*"

woe all "pflege*" and woe all "medika*" and woe all "Geriatri*"

woe all "pflege*" and woe all "medika*" and woe all "Senior*"

woe all "pflege*" and woe all "ausscheid*" and woe all "alt*"

woe all "pflege*" and woe all "Ausscheid*" and woe all "älter*"

woe all "pflege*" and woe all "Essen und Trinken" and woe all "alt*"

woe all "pflege*" and woe all "Essen und Trinken" and woe all "alter*"

woe all "pflege*" and woe all "Essen und Trinken" and woe all "geriatri*"

woe all "pflege*" and woe all "Essen und Trinken" and woe all "senior*"

woe all "pflege*" and woe all "Ernährung*" and woe all "alt*"

woe all "pflege*" and woe all "Ernährung*" and woe all "älter*"

woe all "pflege*" and woe all "Ernährung*" and woe all "geriatri*"

woe all "pflege*" and woe all "Ernährung*" and woe all "senior*"

woe all "pflege*" and woe all "Flüssigkeit*" and woe all "alt*"

woe all "pflege*" and woe all "Flüssigkeit*" and woe all "älter*"

woe all "pflege*" and woe all "Flüssigkeit*" and woe all "geriatri*"

woe all "pflege*" and woe all "Flüssigkeit*" and woe all "senior*"

woe all "pflege*" and woe all "Komfort" and woe all "alt*"

woe all "pflege*" and woe all "Komfort" and woe all "älter*"

woe all "pflege*" and woe all "Komfort" and woe all "geriatri*"

woe all "pflege*" and woe all "Komfort" and woe all "senior*"

woe all "pflege*" and woe all "Zufriedenheit" and woe all "senior*"

woe all "pflege*" and woe all "Zufriedenheit" and woe all "alt*"

woe all "pflege*" and woe all "Zufriedenheit" and woe all "geriatri*"

woe all "pflege*" and woe all "Zufriedenheit" and woe all "älter*"

woe all "pflege*" and woe all "Sicher*" and woe all "alt*"

woe all "pflege*" and woe all "Sicher*" and woe all "älter*"

woe all "pflege*" and woe all "Sicher*" and woe all "geriatri*"

woe all "pflege*" and woe all "Sicher*" and woe all "senior*"

woe all "pflege*" and woe all "Kommunik*" and woe all "alt*"

woe all "pflege*" and woe all "Kommunik*" and woe all "älter*"

woe all "pflege*" and woe all "Kommunik*" and woe all "geriatri*"

woe all "pflege*" and woe all "Kommunik*" and woe all "senior*"

woe all "pflege*" and woe all "interaktion" and woe all "alt*"

woe all "pflege*" and woe all "interaktion" and woe all "älter*"

woe all "pflege*" and woe all "interaktion" and woe all "geriatri*"

woe all "pflege*" and woe all "interaktion" and woe all "senior*"

woe all "pflege*" and woe all "Beteilig*" and woe all "alt*"

woe all "pflege*" and woe all "Beteilig*" and woe all "älter*"

woe all "pflege*" and woe all "Beteilig*" and woe all "geriatri*"

woe all "pflege*" and woe all "Beteilig*" and woe all "senior*"

woe all "pflege*" and woe all "Respekt*" and woe all "alt*"

woe all "pflege*" and woe all "Respekt*" and woe all "älter*"

woe all "pflege*" and woe all "Respekt*" and woe all "geriatri*"

woe all "pflege*" and woe all "Respekt*" and woe all "senior*"

woe all "pflege*" and woe all "Rassismus" and woe all "alt*"

woe all "pflege*" and woe all "Rassismus" and woe all "älter*"

woe all "pflege*" and woe all "Altersdiskriminierung" and woe all "senior*"

woe all "pflege*" and woe all "Altersdiskriminierung" and woe all "alt*"

woe all "pflege*" and woe all "Altersdiskriminierung" and woe all "älter*"

woe all "pflege*" and woe all "Altersdiskriminierung" and woe all "geriatri*"

woe all "pflege*" and woe all "Edukation" and woe all "alt*"

woe all "pflege*" and woe all "Edukation" and woe all "älter*"

woe all "pflege*" and woe all "Edukation" and woe all "geriatri*"

woe all "pflege*" and woe all "Edukation" and woe all "senior*"

woe all "pflege*" and woe all "Information" and woe all "senior*"

woe all "pflege*" and woe all "Information" and woe all "alt*"

woe all "pflege*" and woe all "Information" and woe all "geriatri*"

woe all "pflege*" and woe all "Information" and woe all "älter*"

woe all "pflege*" and woe all "Würde*" and woe all "alt*"

woe all "pflege*" and woe all "Würde*" and woe all "älter*"

woe all "pflege*" and woe all "Würde*" and woe all "geriatri*"

woe all "pflege*" and woe all "Würde*" and woe all "senior*"

woe all "pflege*" and woe all "Wohlbefinden" and woe all "alt*"

woe all "pflege*" and woe all "Wohlbefinden" and woe all "älter*"

woe all "pflege*" and woe all "Wohlbefinden" and woe all "geriatri*"

woe all "pflege*" and woe all "Wohlbefinden" and woe all "senior*"

woe all "pflege*" and woe all "Emotion*" and woe all "senior*"

woe all "pflege*" and woe all "Emotion*" and woe all "alt*"

woe all "pflege*" and woe all "Emotion*" and woe all "geriatri*"

woe all "pflege*" and woe all "Emotion*" and woe all "senior*"

woe all "pflege*" and woe all "Lebensqualität" and woe all "alt*"

woe all "pflege*" and woe all "Lebensqualität" and woe all "älter*"

woe all "pflege*" and woe all "Lebensqualität" and woe all "geriatri*"

woe all "pflege*" and woe all "Lebensqualität" and woe all "senior*"

woe all "pflege*" and woe all "Wert*" and woe all "alt*"

woe all "pflege*" and woe all "Wert*" and woe all "älter*"

woe all "pflege*" and woe all "Wert*" and woe all "geriatri*"

woe all "pflege*" and woe all "Wert*" and woe all "senior*"

woe all "pflege*" and woe all "Überzeugung*" and woe all "alt*"

woe all "pflege*" and woe all "Überzeugung*" and woe all "älter*"

woe all "pflege*" and woe all "Überzeugung*" and woe all "geriatri*"

woe all "pflege*" and woe all "Überzeugung*" and woe all "senior*"

woe all "pflege*" and woe all "Privat*" and woe all "alt*"

woe all "pflege*" and woe all "Privat*" and woe all "älter*"

woe all "pflege*" and woe all "Privat*" and woe all "geriatri*"

woe all "pflege*" and woe all "Privat*" and woe all "senior*"

woe all "pflege*" and woe all "Zuhören" and woe all "senior*"

woe all "pflege*" and woe all "Zuhören" and woe all "alt*"

woe all "pflege*" and woe all "Zuhören" and woe all "geriatri*"

woe all "pflege*" and woe all "Zuhören" and woe all "senior*"

woe all "pflege*" and woe all "Kommunikationsfähigkeit" and woe all "alt*"

woe all "pflege*" and woe all "Kommunikationsfähigkeit" and woe all "geriatri*"

woe all "pflege*" and woe all "empathisch" and woe all "alt*"

woe all "pflege*" and woe all "empathisch" and woe all "älter*"

woe all "pflege*" and woe all "empathisch" and woe all "geriatri*"

woe all "pflege*" and woe all "empathisch" and woe all "senior*"

woe all "pflege*" and woe all "Einfühlungsvermögen" and woe all "alt*"

woe all "pflege*" and woe all "Einfühlungsvermögen" and woe all "geriatri*"

woe all "pflege*" and woe all "Einfühlsam" and woe all "alt*"

woe all "pflege*" and woe all "Achtsam*" and woe all "alt*"

woe all "pflege*" and woe all "Achtsam*" and woe all "älter*"

woe all "pflege*" and woe all "Achtsam*" and woe all "geriatri*"

woe all "pflege*" and woe all "Achtsam*" and woe all "senior*"

woe all "pflege*" and woe all "aufmerksam*" and woe all "alt*"

woe all "pflege*" and woe all "aufmerksam*" and woe all "älter*"

woe all "pflege*" and woe all "aufmerksam*" and woe all "geriatri*"

woe all "pflege*" and woe all "aufmerksam*" and woe all "senior*"

woe all "pflege*" and woe all "Unterstütz*" and woe all "alt*"

woe all "pflege*" and woe all "Unterstütz*" and woe all "älter*"

woe all "pflege*" and woe all "Unterstütz*" and woe all "geriatri*"

woe all "pflege*" and woe all "Unterstütz*" and woe all "senior*"

woe all "pflege*" and woe="sich einlassen" and woe all "senior*"

woe all "pflege*" and woe="sich einlassen" and woe all "alt*"

woe all "pflege*" and woe="Zusammenarbeit mit Patient*" and woe all "alt*"

woe all "pflege*" and woe all "partnerschaft*" and woe all "alt*"

woe all "pflege*" and woe all "partnerschaft*" and woe all "älter*"

woe all "pflege*" and woe all "partnerschaft*" and woe all "geriatri*"

woe all "pflege*" and woe all "partnerschaft*" and woe all "senior*"

woe all "pflege*" and woe all "beruhig*" and woe all "senior*"

woe all "pflege*" and woe all "beruhig*" and woe all "alt*"

woe all "pflege*" and woe all "beruhig*" and woe all "geriatri*"

woe all "pflege*" and woe all "beruhig*" and woe all "älter*"

woe all "pflege*" and woe all "hilf*" and woe all "älter*"

woe all "pflege*" and woe all "hilf*" and woe all "alt*"

woe all "pflege*" and woe all "hilf*" and woe all "geriatri*"

woe all "pflege*" and woe all "hilf*" and woe all "senior*"

woe all "pflege*" and woe all "Bewältig*" and woe all "alt*"

woe all "pflege*" and woe all "Bewältig*" and woe all "älter*"

woe all "pflege*" and woe all "Bewältig*" and woe all "geriatri*"

woe all "pflege*" and woe all "Bewältig*" and woe all "senior*"

woe all "pflege*" and woe all "Mitgefühl" and woe all "senior*"

woe all "pflege*" and woe all "Mitgefühl" and woe all "alt*"

woe all "pflege*" and woe all "Mitgefühl" and woe all "älter*"

woe all "pflege*" and woe all "Mitgefühl" and woe all "geriatri*"

woe all "pflege*" and woe all "Ziel*" and woe all "alt*"

woe all "pflege*" and woe all "Ziel*" and woe all "älter*"

woe all "pflege*" and woe all "Ziel*" and woe all "geriatri*"

woe all "pflege*" and woe all "Ziel*" and woe all "senior*"

woe all "pflege*" and woe all "Pflegeplan" and woe all "alt*"

woe all "pflege*" and woe all "Pflegeplan" and woe all "älter*"

woe all "pflege*" and woe all "Pflegeplan" and woe all "geriatri*"

woe all "pflege*" and woe all "Pflegeplan" and woe all "senior*"

Filters applied: **Limiters**  - Material types: Journals/series, media combinations, books, manuscripts, online resources, electronic data carriers, issues/booklets, articles. Subject groups: 610 Medicine, health, 300 Social sciences, sociology, anthropology. Publication date: 31.08.2024
