# Supplementary material for: Nursing research on physical, relational and psychosocial care for older people in Germany: protocol for a mapping review guided by the Fundamentals of Care Framework
Source: Syst Rev. 2026 May 8;15:160. doi: 10.1186/s13643-026-03191-0 (PMC13154650; doi:10.1186/s13643-026-03191-0)
Supplement: Supplementary file 3 — Additional file 3: Data charting form. [file 13643_2026_3191_MOESM3_ESM.docx]

**Additional form 3: The data charting/extraction form**

- First author
- Discipline and/or profession and organisation of the researcher undertaking the basic research in nursing
- Year of publication
- Aim of the study
- Research questions
- Study designs such as cross-sectional studies, randomized controlled trials, qualitative studies (e.g., grounded theory, phenomenology), and systematic reviews.
- Research methods such as quantitative, qualitative, mixed-methods
- Measurements
- Study population, including number, sex and age of participants
- Setting of the study
- Place of the study
- Period of the study
- Type of research, e.g. qualification work such as original study, dissertation, master's thesis)
- Funding status (if funded, specify the funding institution)
- Language of the study (e.g., German or English)
- Reported outcomes or phenomena of interest of nursing research studies focusing on older people in Germany, and classification according to;

1. The physical (*subcomponents: Personal cleansing and dressing, toileting, eating and drinking, rest and sleep, mobility, comfort, safety, and medication management*),
2. Psychosocial (*subcomponents:* *Communication, being kept informed and involved, privacy, dignity, respect, education and information provision, emotional well-being, and consideration of values and beliefs)*, and
3. Relational care actions (*subcomponents:* *Active listening, empathy, engagement, compassion, presence, involving family and carers, supporting coping, goal-setting and evaluation with patients, and helping patients remain calm*) components and subcomponents of the Integration of Care dimension of the FOC framework.

- Main results and conclusions
