# Supplementary material for: Nursing research on physical, relational and psychosocial care for older people in Germany: protocol for a mapping review guided by the Fundamentals of Care Framework
Source: Syst Rev. 2026 May 8;15:160. doi: 10.1186/s13643-026-03191-0 (PMC13154650; doi:10.1186/s13643-026-03191-0)
Supplement: Supplementary file 4 — Additional file 4: Coding Framework for Data Classification (FOC-Based). [file 13643_2026_3191_MOESM4_ESM.docx]

**Additional form 4: Coding Framework for Data Classification (FOC-Based**

This coding framework operationalises the Integration of Care dimension of the Fundamentals of Care (FOC) framework for the systematic classification of included studies. Coding will be conducted in two complementary stages, based on the framework’s predefined components and subcomponents as defined by Kitson et al. (2010), Feo, Kitson and Conroy (2018), and Feo et al. (2025) (1–3). An initial classification based on study aims and/or research questions, followed by a refinement stage based on reported measurement-based outcomes and phenomena of interest. In the initial phase, studies will be coded if their aims and/or research questions align with any component or subcomponent of the Integration of Care dimension of the FOC framework. In the secondary phase, studies will be further coded based on measurement-based outcomes and/or phenomena of interest, provided that these also align with at least one study aim and/or research question. A study will only be assigned to a component when its aims and/or research questions (where available) demonstrate alignment with at least one subcomponent of the Integration of Care dimension of the FOC framework. This two-step process ensures consistency between study objectives, research questions, and empirically measured and reported outcomes/phenomena of interest, as well as anticipated outcomes and phenomena of interest in study or review protocols.

Multiple component/subcomponent coding will be applied where studies address more than one component. Each included study will be assigned to one or more of the following components:

**A. Coding Components and Decision Rules**

**1. Physical Care Recipient Needs**

Studies will be coded under the physical care component if their primary focus relates to the physical care needs of older people, focusing on the following outcomes and/or phenomena of interest within the subcomponents below:

- rest and sleep
- personal cleansing and dressing
- medication management
- toileting needs
- eating and drinking
- comfort
- safety
- mobility

**Coding rule:** Assigned when the study aim, research questions (where available), and/or measurements based reported outcomes and/or phenomena of interest relate to at least one physical care need (e.g., mobility, nutrition, hygiene, medication, sleep, comfort, safety).

**2. Psychosocial Care Recipient Needs**

Studies will be coded under the psychosocial care component if their primary focus relates to psychosocial care needs of older people, focusing on the following outcomes and/or phenomena of interest within the subcomponents below:

- communication
- being involved and informed
- respect
- dignity
- education and information
- having values and beliefs considered and respected
- emotional wellbeing
- privacy

**Coding rule:** Assigned when the study aim, research questions (where available), and/or measurements based reported outcomes relate to psychosocial experiences, perceptions, communication, dignity, emotional well-being, values, or related aspects of care.

**3. Relational Caregiver Actions**

Studies will be coded under the relational care action component if their primary focus concerns relational care actions for older people, focusing on the following outcomes and/or phenomena of interest within the subcomponents below:

- being empathetic
- helping patients to cope
- engagement with patients
- supporting and involving families and carers
- working with patients to set goals
- active listening
- helping patient to stay calm
- being compassionate
- being present

**Coding rule:** Assigned when the study aim, research questions (where available), and/or measurements based reported outcomes and/or phenomena of interest relate to healthcare professionals’ relational behaviours, such as empathy, communication, engagement, support, presence, or collaboration.

**B. Multi-Component Coding Rule**

Studies addressing more than one component (e.g., physical + psychosocial) will be assigned to **all relevant components**.

No hierarchical prioritisation is applied; all relevant components are coded.

**C. Unit of Coding**

- The unit of analysis is the individual study
- Coding is based on the dominant focus of the study, determined by:
  - stated aim
  - research questions (if available)
  - measurement based reported outcomes or phenomena of interest

Where ambiguity exists, consensus discussion between reviewers will determine final classification.

**D. Use of Subcomponents**

FOC-Integration of Care subcomponents were used as analytical references to support coding at the component level (physical, psychosocial, and relational), and were additionally applied where sufficient detail was available to allow more precise classification within each component.

**References**

1. Feo R, Kitson A, Conroy T. How fundamental aspects of nursing care are defined in the literature: A scoping review. J Clin Nurs. 2018;27(11–12):2189–229.

2. Kitson A, Conroy T, Wengstrom Y, Profetto-McGrath J, Robertson-Malt S. Defining the fundamentals of care. Int J Nurs Pract. 2010;16(4):423–34.

3. Feo R, Conroy T, Laugesen B, Voldbjerg SL, Gronkjaer M, Jensen K. Assessing the Complexity of Fundamental Care : Developing and Refining the Flinders Fundamentals of Care Assessment Tool for Clinical Practice. 2025;8972–89.
